# N-Terminal Deletion of Peptide:*N*-Glycanase Results in Enhanced Deglycosylation Activity

**DOI:** 10.1371/journal.pone.0008335

**Published:** 2009-12-16

**Authors:** Shengjun Wang, Fengxue Xin, Xiaoyue Liu, Yuxiao Wang, Zhenyi An, Qingsheng Qi, Peng George Wang

**Affiliations:** 1 State Key Laboratory of Microbial Technology, Shandong University, Jinan, China; 2 National Glycoengineering Research Center, Shandong University, Jinan, China; Institut Europeen de Chimie et Biologie, France

## Abstract

Peptide:*N*-glycanase catalyzes the detachment of N-linked glycan chains from glycopeptides or glycoproteins by hydrolyzing the β-aspartylglucosaminyl bond. Peptide:*N*-glycanase in yeast binds to Rad23p through its N-terminus. In this study, the complex formed between Peptide:*N*-glycanase and Rad23p was found to exhibit enhanced deglycosylation activity, which suggests an important role for this enzyme in the misfolded glycoprotein degradation pathway *in vivo*. To investigate the role of this enzyme in this pathway, we made stepwise deletions of the N-terminal helices of peptide:*N*-glycanase. Enzymatic analysis of the deletion mutants showed that deletion of the N-terminal H1 helix (Png1p-ΔH1) enhanced the deglycosylation activity of *N*-glycanase towards denatured glycoproteins. In addition, this mutant exhibited high deglycosylation activity towards native glycoproteins. Dynamic simulations of the wild type and N-terminal H1 deletion mutant implied that Png1p-ΔH1 is more flexible than wild type Png1p. The efficient deglycosylation of Png1p-ΔH1 towards native and non-native glycoproteins offers a potential biotechnological application.

## Introduction

In eukaryotes, newly synthesized proteins, which are destined for the secretory pathway, are subjected to a quality control system [1], [2]. In this control system, the proteins that fail to fold correctly are retained in the ER and subsequently degraded by a mechanism known as “ER-associated degradation” (ERAD) [3]. Peptide:*N*-glycanase (PNGase, PNGase from yeast was also named Png1p) is an important enzyme involved in this ERAD pathway and contributes to the degradation of misfolded N-linked glycoproteins[4], [5], [6]. PNGase catalyzes the detachment of N-linked glycan chains from glycopeptides or misfolded glycoproteins by hydrolyzing the β-aspartylglucosaminyl bond [5].

*In vivo*, PNGase binds to the 26S proteasome through its interaction with a component of the DNA repair system, Rad23p, which is known to play a pivotal role in nucleotide excision repair [7], [8], [9], [10]. Rad23p contains four structural domains connected by long unstructured flexible linker regions: an ubiquitin-like domain (UBL) at the N terminus that interacts with catalytically active proteasomes, two ubiquitin (Ub)-associated (UBA) sequences that bind Ub and a XPC binding (XPCB) domain that mainly mediates the interaction with PNGase. The surface of the XPCB domain of Rad23p is almost entirely hydrophobic, suggesting that Rad23p interacts with particular proteins via hydrophobic interactions [11]. Crystal structure analysis of the Rad23p-PNGase complex revealed that Rad23p forms extensive interactions with both the N- and C-terminal helices of PNGase [12]. Biochemical studies have shown that the H1α-helix at the N-terminus of PNGase is mainly composed of hydrophobic amino acids. A recent report showed that PNGase with an extended N-terminus in mammalian cells interacts with another protein, Derlin-1, via its N-terminal domain [13], [14], [15]. In mice, a complex containing five proteins, mAMFR, mY33K, mp97, mPNGase and mHR23B is formed in close proximity to the ER membrane [8]. The formation of a stable complex between PNGase and Rad23p was suggested to open up the conformation of Rad23p, which in turn facilitates the binding of Rad23p to the proteasome and/or ERAD substrates [16]. However, is there any structural change to PNGase upon binding Rad23p and what role does this interaction have to PNGase activity?

The crystal structure and biochemical analysis confirmed that three amino acids, Cys-191, His-218 and Asp-235, are crucial in PNGase catalysis [5], [12], [17]. This catalytic triad is located in a “transglutaminase” motif which contributes to degradation of glycoproteins and represents one of the most conserved regions among PNGase from various organisms. Therefore, PNGase have been proposed to be part of the transglutaminase-like superfamily [18]. Besides the deglycosylation activity, PNGase from *C. elegans* also exhibits oxidoreductase (thioredoxin) activity, suggesting PNGase play an important role in higher eukaryotes [19]. Since the catalytic center of PNGase from yeast is located in the central part of the enzyme, and distal from the terminal domains, it is likely that both the N-terminus (H1, H2) and the C-terminus (H11, H12) regions provide binding motifs with other proteins and are not essential for PNGase activity [12]. Park *et al*, revealed that the N-terminal domain and the middle domain of mammalian PNGase is important for the deglycosylation activity. In this report, it was demonstrated that a PNGase C-terminal deletion mutant (amino acids 1–471) or full-length PNGase exhibited deglycosylation activity, whereas a PNGase N-terminal deletion mutant (amino acids 171–651), and the PNGase core region mutant (middle domain, amino acids 171–471) exhibited no detectable activity [8], [9]. However, the N-terminal H1 and H2 helices of yeast Png1p are absent in the mammalian enzyme. A recent study found that the C-terminal domain of mouse PNGase binds to the mannose moieties of N-linked oligosaccharide chains and enhances the activity of the core domain, presumably by increasing the affinity of mouse PNGase for the glycan chains of misfolded glycoproteins [20]. In yeast, Png1p does not possess a separate C-terminal domain and therefore does not binds to mannopentaose; however, a separate binding site for chitobiose and other carbohydrates exists [21], [22], [23].

PNGase distinguishes native and non-native glycoproteins *in vitro* [13], [24]. PNGase can not deglycosylate correctly folded native glycoproteins, but catalyzes the deglycosylation of misfolded glycoproteins. Our experiments found that the overall deglycosylation activity of Png1p from yeast is lower than a commercially available deglycosylation enzyme PNGase F from *Flavobacterium meningosepticum*, which is extensively used as a biochemical tool for the study and analysis of glycoproteins [25]. This enzyme is an alkaline enzyme with optimal activity at pH 8.5. In this study, we analyzed the interaction of PNGase and Rad23p from yeast *in vitro* and identified that the N-terminus was functionally important. Stepwise deletions of the terminal helices of PNGase were performed to further analyze the functional roles of the terminal residues. The properties of the deletion mutants were analyzed.

## Results

### Interaction between Png1p and Rad23p Increases the Deglycosylation Activity of Png1p

**Figure 1:**
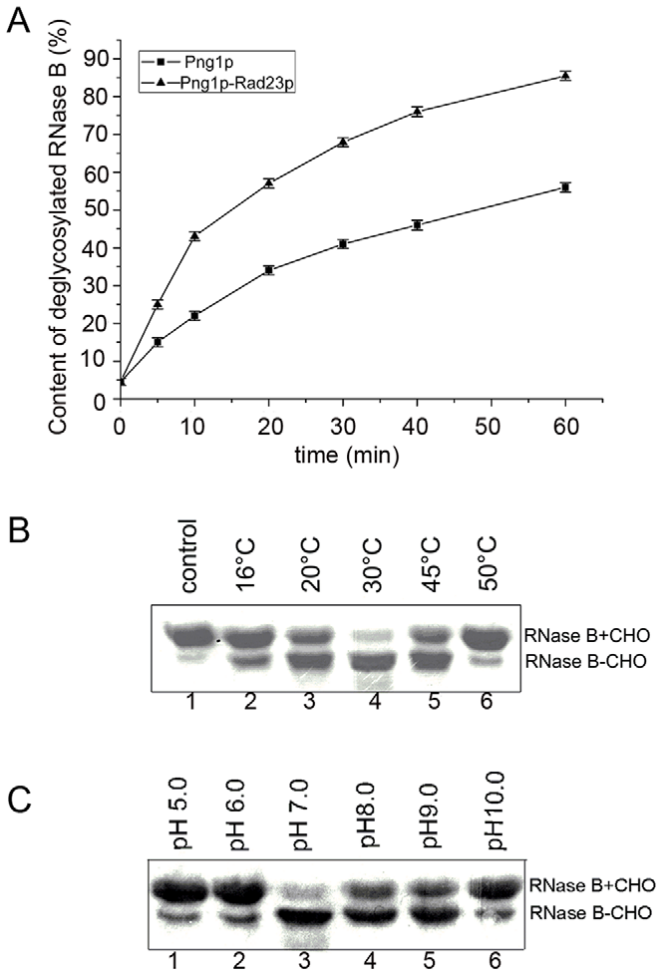
Enzymatic properties of Png1p-Rad23p complex. (**A**) Comparison of deglycosylation activity between Png1p and Png1p-Rad23p complex. Png1p and Png1p-Rad23p complex were incubated with RNase B (0.5 mg/ml) in 30 µl of 50 mM Hepes buffer, pH 7.0, and 5 mM DTT at 30°C. (**B**) Influence of temperature on Png1p-Rad23p complex activity. Png1p-Rad23p complex was incubated with denatured RNase B (0.5 mg/ml) in 30 µl of 50 mM Hepes buffer, pH 7.0 at different temperature for 1 h. (**C**) Influence of pH on Png1p-Rad23p complex activity. Png1p-Rad23p complex was incubated with denatured RNase B (0.5 mg/ml) in 30 µl buffer with different pH at 30°C for 1 h. (pH 5.0–8.0, Na_2_HPO_4_- Citric Acid; pH 9.0 and pH 10.0, Gly-NaOH). All proteins used in the assay were purified at the same time, following the same protocol. The molar ratio of enzyme to substrate was 1∶30 in each reaction. Samples were taken at the indicated time points and subjected to SDS–PAGE followed by Coomassie staining. The zero time point was taken prior to addition of Png1p-Rad23p complex. The resulting Coomassie stained gels were quantified by densitometry with Image J program.

Both *in vitro* and *in vivo*, Png1p interacts strongly with Rad23p through its N-terminal region [7], [8], [9], [10]. *In vivo*, the Png1p-Rad23p interaction facilitates the direct transfer of deglycosylated ERAD substrates to the proteasome, which bind to the UBL domain of Rad23p. While the interaction of Png1p and Rad23p leads to the opening of the Rad23p conformation, [16] there is a paucity of data defining any structural changes to Png1p upon interaction with Rad23p. We thus prepared the Png1p-Rad23p complex according to the method of Biswas *et al* [26] and examined the deglycosylation activity of the Png1p-Rad23p complex *in vitro*. We found that the deglycosylation activity of the Png1p-Rad23p complex was enhanced compared with the activity of Png1p only (Fig. 1A). Under the same reaction conditions, 40% more glycoproteins were deglycosylated by the Png1p-Rad23p complex. In a control experiment, Rad23p was observed to have no deglycosylation activity. This finding indicated that the N-terminus of Png1p mediated Png1p-Rad23p interaction *in vivo* benefits not only Rad23p activity but also enhances the deglycosylation activity of Png1p. Enhanced deglycosylation activity may accelerate the degradation of misfolded glycoproteins when they are translocating through the ER membrane and therefore eliminates the accumulation of these misfolded glycoproteins.

The stability of the Png1p-Rad23p complex was examined. The Png1p-Rad23p complex showed higher stability than Png1p. Png1p was inactive at 37°C (Figure S3). In contrast, the Png1p-Rad23p complex still possessed enzymatic activity at 45°C (Fig. 1B). The complex also exhibited a broad pH adaptation, from pH 5.0 to 10.0 (Fig. 1C). The optimum deglycosylation temperature and pH of the Png1p-Rad23p complex was 30°C and pH 7.0, which is similar to Png1p alone. The results indicate that the Png1p-Rad23p complex played a main role in deglycosylation, while cytosolic free Png1p supplements this process.

### Structural Analysis and Molecular Simulation of Png1p

**Figure 2:**
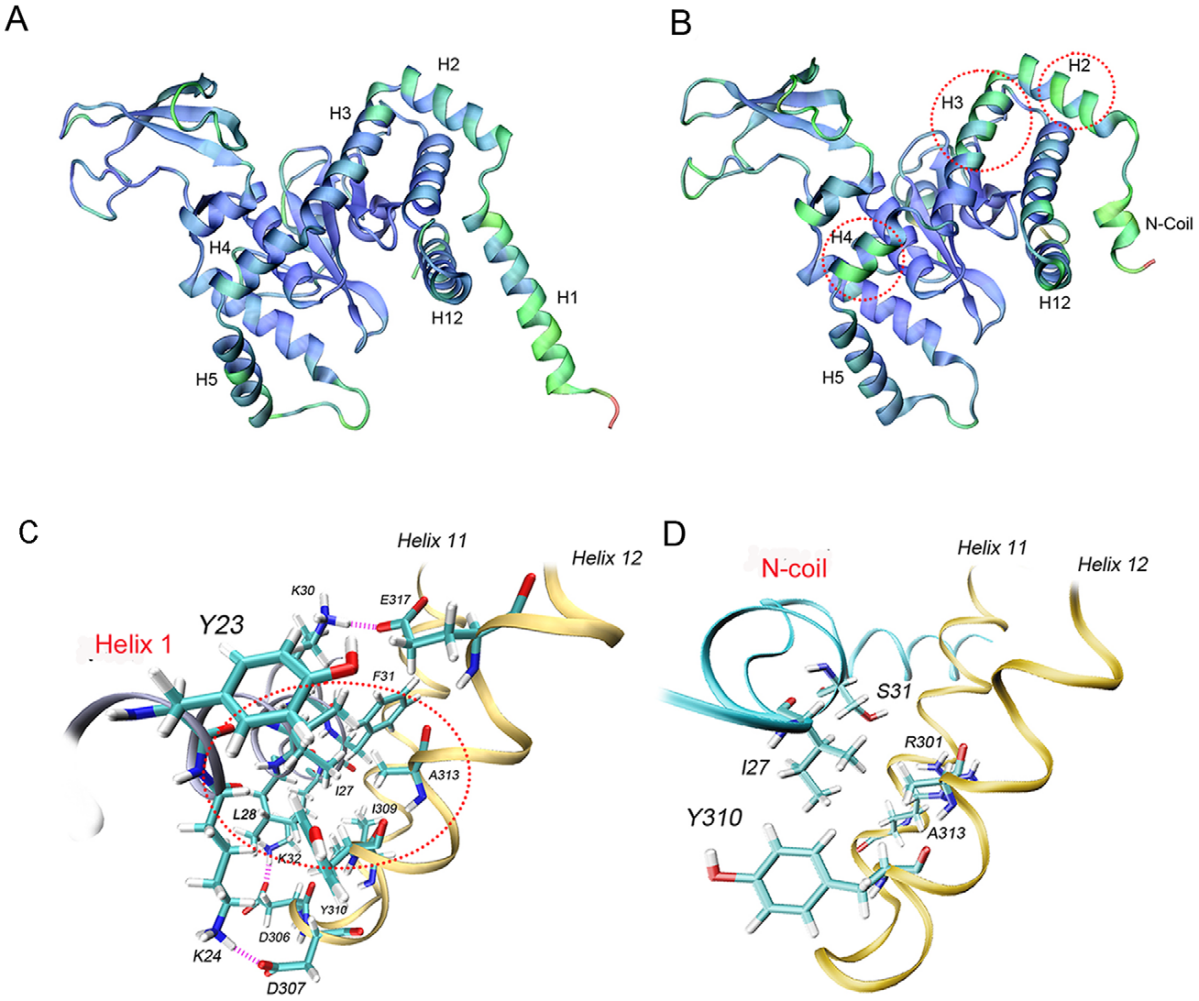
Molecular model of full-length Png1p and deletion mutant Png1p-ΔH1. (**A**) Average structure of Png1p. (**B**) Average structure of Png1p-ΔH1. Each structure is an average structure from the final 200 ps of each simulation. The structure is colored by RMSD from blue (lowest RMSD) to green and then red (highest RMSD). Only N-terminal residue is colored red because of its especial high flexibility. Parts of the protein with increased flexibility are marked as red circle. (**C**) Close-up view of the interaction between helices H11, H12 (*orange*) and N-terminal helix H1. (**D**) Close-up view of the interaction between helices H11, H12 (*orange*) and N-terminal coil. Residues involved in hydrogen bonds are shown in *green* and the interacting helices are labeled. Lys 24, Lys 30, and Lys 32 on H1 helix of Png1p continuously interact with Asp 307, Glu 317 and Asp 306 on H12 helix, while only Ser 31 on N-terminal coil of Png1p-ΔH1 hydrogen bonds to residue Arg 301. Hydrogen bonds are shown as magenta dashed line. Hydrophobic cluster is indicated by red circle.

The crystal structure of Png1p-Rad23p complex has been solved [12]. Analysis of the structure revealed that the N-terminal H1 helix of yeast Png1p is extended away from the core domain and absent in the mammalian enzyme [21], [22], [23]. Consequently, this observation indicates that the N-terminal H1 helix is not directly involved in catalysis. To understand the structural basis of Png1p and the role of the N-terminus, a molecular model of Png1p was constructed based on this crystallographic structure [1X3W] (Fig. 2A, C). In the model, helices H2 and H3 located on the top of the active site cleft may inhibit the correct positioning of the native substrate into the active site. The interaction of H1 with Rad23p may displace helices H2 and H3 from the active site cleft (Fig. 2A). Molecular simulations of the last 200 ps were performed (Figure S4). We found that residues Lys 24, Lys 30 and Lys 32 located within the N-terminal helix H1 continuously interacted with residues Asp 307, Glu 317 and Asp 306 located within helix H12, respectively (Fig. 2C). These charged residues form strong electrostatic interactions and may act as a type of “electrostatic glue” thereby fixing the rear part of helix H1 on to helix H12. In addition, a dense hydrophobic cluster was formed by the side-chains of Ile27, Leu28 and Phe31 on helix H1 and Ile 309, Tyr 310 and Ala 313 on helix H12. Hydrophobic residues on helix H1 interacted extensively with non-polar side-chains on helix H12, which may further stabilize the relative position of helix H1 and helix H12.

### An N-Terminal H1 Deletion Mutant Shows Enhanced Deglycosylation Activity

**Figure 3:**
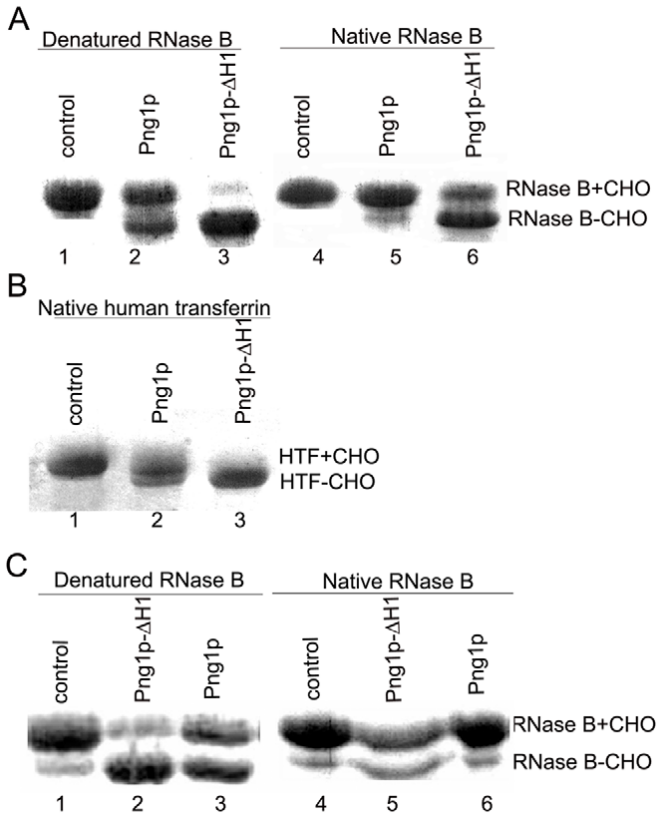
Enzymatic properties of Png1p-ΔH1. (**A**) Deglycosylation of RNase B by Png1p or Png1p-ΔH1 from *S. cerevisiae.* (**B**) Deglycosylation of Human transferrin (HTF) by Png1p-ΔH1 from *S. cerevisiae;* (**C**) Deglycosylation of RNase B by Png1p or Png1p-ΔH1 from *S. pombe.* Png1p and Png1p-ΔH1 were incubated with RNase B (0.5 mg/ml) or HTF (1 mg/ml) in 30 µl of 50 mM Hepes buffer, pH 7.0 and 5 mM DTT at 30°C for 1 h. All proteins used in the assay were purified at the same time, following the same protocol. The molar ratio of enzyme to substrate was 1∶30 in each reaction. After enzymatic digestion, samples were analyzed by SDS–PAGE followed by Coomassie staining. +CHO represents the glycosylated form of protein, and -CHO represents the deglycosylated form of protein.

To characterize the function of the N-terminal H1 helix, we constructed an N-terminal deletion mutant, Png1p-ΔH1 (33–363 aa). Biochemical analysis showed that the Png1p-ΔH1 mutant was unable to form a stable complex with Rad23p, which is consistent with the previous result that the N-terminus of Png1p is responsible for protein-protein interactions [26]. Interestingly, we found Png1p-ΔH1 exhibited a remarkable increase in deglycosylation activity on denatured glycoproteins when compared with the activity of native Png1p (Fig. 3A). Moreover, we also found that the N-terminal deletion mutant acted both on non-native and native glycoproteins *in vitro*; whereas wild type Png1p acted only on misfolded glycoproteins. This is an exciting finding because Png1p from yeast has been shown to distinguish between native and non-native glycoproteins *in vitro* [24]. Deglycosylation of non-native glycoproteins by Png1p is an important quality control process in the ERAD pathway [13], [24]. Recognition of native glycoprotein substrates by Png1p-ΔH1 aids in our efforts to unravel the reaction mechanism of the enzyme and facilitates potential biotech applications. To further characterize the deglycosylation activity of Png1p-ΔH1 towards native proteins, native human transferrin (HTF), which bears a complex asparagine-linked oligosaccharide, was employed [27]. Experimental results revealed that Png1p-ΔH1 was also able to deglycosylate HTF (Fig. 3B). We then constructed a series of PNGase deletion mutations, Png1p-ΔH12, Png1p-ΔH1H12 and Png1p-ΔH1H2H11H12, to characterize the function of other regions of the protein. None of these mutants showed deglycosylation activity *in vitro*. To see if the N-terminal deletion of peptide: *N*-glycanase from other organisms also exhibited these properties, we performed the same experiment with Png1p from *Schizosaccharomyces pombe*, a recently characterized peptide: *N*-glycanase [28]. The deglycosylation activity of Png1p-ΔH1 from *S. pombe* was also enhanced, acting on both denatured and native glycoproteins (Fig. 3C).

### Possible Role of the N-Terminal H1 Helix in Png1p

**Figure 4:**
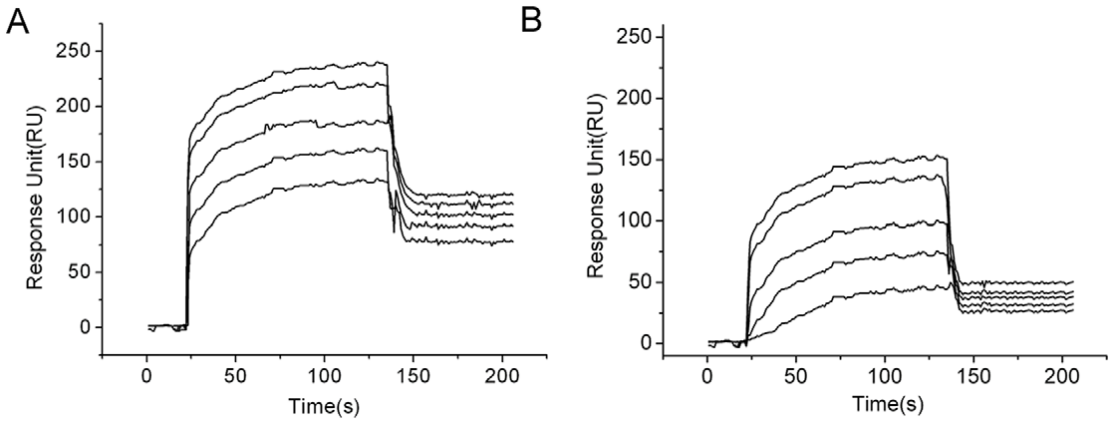
Kinetic analysis of Png1p and Png1p-ΔH1 binding to RNase B. RNase B was covalently attached to CT405 chip via their -NH_2_, Different concentrations of Png1p-ΔH1(A panel) and Png1p(A panel) were passed over the chip surface, and kinetics of binding was monitored as response units (RU, y axis) on time (seconds, x axis). Concentrations of Png1p-ΔH1 applied were 80 nM, 320 nM, 640 nM, 800 nM and 1000 nM; Png1p were 386 nM, 344 nM, 688 nM, 869 nM and 1000 nM. The data obtained were analyzed with the SPRViewer software (version 1.0) provided by the manufacturer. Png1p-ΔH1 proved to possess higher binding capability for RNase B than Png1p.

The ability to distinguish native and non-native glycoproteins is an important feature of peptide: *N*-glycanase, which contributes to the quality control function towards newly synthesized glycoproteins in eukaryotes. Deletion of the N-terminal H1 helix abolished the ability of this protein to distinguish between native and misfolded proteins. We compared the binding capability of RNase B to Png1p-ΔH1 with that of Png1p. The binding kinetics for the interactions of Png1p/Png1p-ΔH1 with denatured RNase B was analyzed by surface plasmon resonance (Fig. 4 and Table 1). The results indicated that RNase B bound more strongly to Png1p-ΔH1 (K_D_ = 2.9×10^−6^ M) in comparison with wild type Png1p (K_D_ = 7.7×10^−6^ M).

**Table 1:** Kinetics analysis of interaction between PNGase and RNase B using SPR.

	k_on_ (M^−1 ^s^−1^)	k_off_ (s^−1^)	K_D_(M)
Png1p	1.22×10^4^	9.4×10^−2^	7.7×10^−6^
Png1p-ΔH1	1.7×10^4^	5×10^−2^	2.9×10^−6^

Kinetics values of the interaction of Png1p/Png1p-ΔH1 with Denatured RNase B were calculated using 1∶1 Langmuir model. Association rate (k_on_), dissociation rate (k_off_), and dissociation constants (K_D_ = k_off_/k_on_) are given.

We then compared the average dynamic structure of the last 200 ps by molecular simulation (Figure S4). Calculation of the Cα RMS deviation (RMSD) for residues 33 to 62 (H2 and H3 helices) over the last 200 ps showed that RMSD increased from 0.1251 to 0.1715, which indicates that the flexibility of helices H2, H3 and H4 of the protein increased following the deletion of the H1 helix (Table S1). The RMSD per residue graph also showed that H2 and H3 helices were more flexible when helix H1 was deleted (Figure S5). This result indicates that the N-terminal deletion may increase enzyme activity. Additionally, Png1p-ΔH1 lacks the electrostatic interaction or hydrophobic center, resulting in weak interactions between helix H1 and helix H12, thereby increasing the flexibility of Png1p-ΔH1 (Fig. 2B, D).

### Png1p-ΔH1 Has a Potential Biotechnological Application

**Figure 5:**
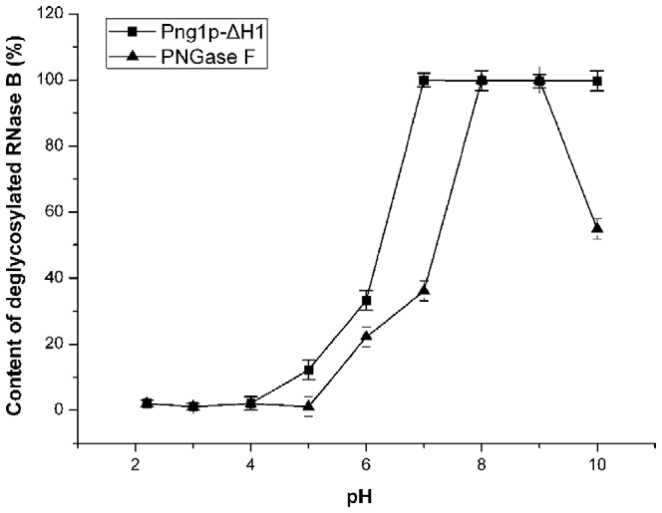
Comparison of deglycosylation activity between Png1p-ΔH1 and PNGase F at different pH. Png1p-ΔH1 was incubated with denatured RNase B (0.5 mg/ml) in 30 µl buffer with different pH at 30°C for 1 h while PNGase F was incubated at 37°C for 1 h at the same condition. (pH 2.0–8.0, Na_2_HPO_4_- Citric Acid; pH 9.0 and pH 10.0, Gly-NaOH). All proteins used in the assay were purified at the same time, following the same protocol. The molar ratio of enzyme to substrate was 1∶30 in each reaction. Samples were taken at the indicated time points and subjected to SDS–PAGE followed by Coomassie staining. The zero time point was taken prior to addition of enzyme. The resulting Coomassie stained gels were quantified by densitometry with Image J program.

Since Png1p-ΔH1 exhibited high deglycosylation activity, we compared this activity with that of the commercially available deglycosylation enzyme, PNGase F. The results showed that Png1p-ΔH1 has a wider pH adaptation (Fig. 5). Even at pH 10.0, Png1p-ΔH1 still retains almost 100% enzymatic activity. Moreover, Png1p-ΔH1 exhibited higher activity under neutral conditions. The results indicate that Png1p-ΔH1 has potential biotechnological applications, especially at neutral pH.

**Figure 6:**
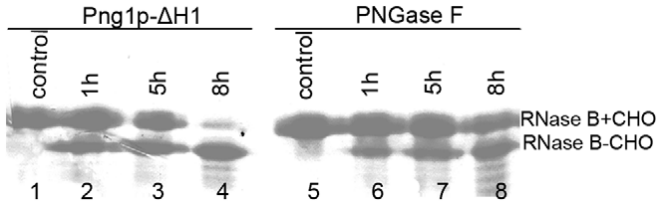
In-gel deglycolation by PNGase F or Png1p-ΔH1 digestion. PNGase F or Png1p-ΔH1 was incubated with RNase B in gel in 30 µl of 20 mM pH 7.0 NaHCO_3_. All proteins used in the assay were purified at the same time, following the same protocol. The molar ratio of enzyme to substrate was 1∶30 in each reaction. After enzymatic digestion, samples were analyzed by SDS–PAGE followed by Coomassie staining. +CHO represents the glycosylated form of protein, and -CHO represents the deglycosylated form of protein.

To confirm this, we performed a deglycosylation analysis experiment using an in-gel PNGase digestion assay [29]. This method is often used to extract oligosaccharides of the glycoprotein that have been separated by SDS-PAGE or 2D-PAGE and subsequently sent for structural analysis by MALDI-TOF mass spectrometry. Digestion with commercial PNGase F at pH 7.0 (the neutral pH is necessary for subsequent experiments) usually takes 16–24 h to release the oligosaccharides from the glycoproteins [29], [30], [31], [32]. The same reaction with Png1p-ΔH1 required only 8 h (Fig. 6).

## Discussion

Peptide:*N*-glycanase is a deglycosylating enzyme that has been suggested to be linked to proteasome-dependent degradation of misfolded glycoproteins translocated from the ER to the cytosol [33]. Peptide:*N*-glycanase plays a key role in the degradation of a subset of glycosylated ERAD substrates. A retrotranslocated, misfolded glycoprotein is first deglycosylated by peptide:*N*-glycanase and subsequently degraded by the proteasome [34], [35]. In yeast, peptide:*N*-glycanase (often named Png1p) binds to the 26S proteasome through its interaction with a component of the DNA repair system, Rad23p, which is known to have a pivotal role in nucleotide excision repair [7], [8], [9], [10].

*In vivo*, the Rad23p-Png1p complex directly couples protein deglycosylation with proteasome degradation in the cytoplasm, thereby ensuring rapid turnover of misfolded glycoprotein and efficient proteasome degradation. At the molecular level, the interaction of Png1p and Rad23p leads to the opening of the conformation of Rad23p, which in turn facilitates its binding to the proteasome and/or ERAD substrates [16]. Since no crystal structure of Png1p has been resolved, we have no information on the possible conformational changes that occur to Png1p upon binding to Rad23p; however, this interaction must play a role in Png1p function. Here, using a biochemical method, we demonstrated *in vitro* that the interaction of Png1p-Rad23p enhanced the deglycosylation efficiency of Png1p. The rapid deglycosylation of misfolded glycoproteins by the Png1p-Rad23p complex may ensure the efficient and direct transfer of deglycosylated ERAD substrates to the proteasome. A previous *in vivo* study also suggested that efficient degradation of glycosylated RTA (ricin A chain) requires the association of Png1p and Rad23p [16]. The half-life of glycoproteins in Rad23p mutant cells is longer. Consequently, both Png1p and Rad23p most likely benefit from the formation of a complex between the two proteins. The Png1p-Rad23p complex was observed to be thermally more stable than Png1p. Free Png1p in the cytosol may deglycosylate the glycoprotein fragments generated by proteolysis or unfolded glycoproteins by reducing conditions maintained in cytosol. Molecular simulations indicated that when Png1p binds to Rad23p, those residues at the contact surface experience a slight chemical shift because of the altered chemical environment. This interaction may release this region from the core domain leading to greater flexibility of the core region. We suggest that such conformational changes upon Png1p-Rad23p complex formation play a central role in regulating the degradation efficiency of misfolded glycoproteins.

The complex interface of the yeast Png1p-Rad23p complex is fundamentally different from the orthologous mammalian peptide N-glycanase-Rad23 complex. The N-terminal H1 and H2 helices of yeast Png1p are absent in the mammalian enzyme [21], [22], [23]. As such, it is plausible that the N-terminus (H1, H2) is not essential for the activity of PNGase [12]. To characterize this postulate, the N-terminal helices of Png1p, which are mainly responsible for protein interactions, were deleted. Interestingly, the deletion mutant Png1p-ΔH1 showed a significant increase in deglycosylation activity when compared with the activity of the wild type enzyme. Moreover, Png1p-ΔH1 was found to act not only on denatured glycoprotein, but also on native glycoproteins *in vitro*. Peptide:*N*-glycanase was previously confirmed to distinguish between native and non-native glycoprotein *in vitro* [13], [24]. However, the exact mechanism of this function is unknown. The observed high deglycosylation activity of Png1p-ΔH1 towards native glycoprotein implies that it can be used as an important model for investigating the deglycosylation mechanism and has potential applications in the biotechnology sector.

Molecular simulations of Png1p-ΔH1 were performed to interpret the observed N-terminal deletion phenomenon. RMSD results showed that the electrostatic and hydrophobic interactions between helices H1 and H12 could stabilize helices H2, H3 and H4, which stabilizes the protein fold (i.e. increase in rigidity)and is not suitable for the positioning of large native glycoproteins (Fig. 2C). The flexibility of helices H2, H3 and H4 was found to be considerably increased following the deletion of helix H1. As a result, the originally narrow active site cleft could gradually be enlarged by the interaction forces between Png1p-ΔH1 and the substrate, thus allowing substrates to access the deep active site more easily. Based on these findings, we hypothesize that the contact of Png1p with a glycoprotein substrate, especially the glycan part of the substrate, will induce a conformational change of the enzyme. This indicates that Png1p will tend to adopt an open conformation. Conversely, the electrostatic and hydrophobic interactions between H1 and H12 helices restrict protein flexibility, therefore inhibiting the formation of an open conformation. Thus, only small polypeptides or denatured glycoproteins can slide into the narrow active site cleft. Deletion of H1 helix provides additional flexibility to the enzyme thereby allowing the enzyme to adopt a more open conformation for interaction with both native and non-native glycoproteins.

Png1p-ΔH1 can be applied in many biological areas, especially where PNGase F is not efficient. Successful application in in-gel deglycosylation experiments for subsequent MALDI-TOF mass spectrometry analysis using Png1p-ΔH1 represents an initial application. The deglycosylation of native glycoproteins by Png1p-ΔH1 may represent a more useful routine application over current methods

## Materials and Methods

### Construction of Plasmids

DNA manipulations were performed according to the instruction of the manufacturer. Both *Png1* and *Rad23* genes used in this study were isolated from the genomic DNA of *S. cerevisiae* W303-1a by polymerase chain reaction (PCR) using *pfu* polymerase (Fermentas, MBI,Canada). Png1p deletion mutants (amino acids 1–363, amino acids 33–363, amino acids 33–305, amino acids 50–286) were obtained by PCR reactions employing the primers having a 5′-*Eco*RI site and a 3′-*Hin*dIII site. The primers are summarized in Table S2. The corresponding PCR sequences were cloned into the *Eco*RI and *Hin*dIII sites of vector pET-22b (+) (Novagen, Carlsbad, USA) generating plasmids pET-22b/*png1p*, pET-22b/*png1p-*Δ*H1*, pET-22b/*png1p-*Δ*H12*, pET-22b/*png1p-*Δ*H1H12*, and pET-22b/*png1p-*Δ*H1 H2 H11 H12*, respectively (Figure S1). The *Rad23* gene was cloned into the *Nde*I and *Bam*HI sites of the pET-15b (+) vector (Novagen, Carlsbad, USA) generating plasmid pET-15b/*Rad23p*. All the resulting constructs were confirmed by DNA sequencing.

### Bacterial Expression and Purification of the Full-Length, Truncated Png1p, PNGase F and Full-Length Rad23p

Plasmids pET-22b/*png1p*; pET-22b/*png1p-*Δ*H1*; pET-22b/*png1p-*Δ*H1H12;* pET-22b/*png1p-*Δ*H1H2H11H12* and pET-15b/*Rad23p* were transformed into *E.coli* BL21 (DE3) pLysS, repectively. Expression of these constructs in *E.coli* BL21 (DE3) pLysS was performed in LB medium by adding 1 mM IPTG at OD_600_ = 0.8/ml. After 4 h of induction at 30°C, cells were harvested and disrupted using sonication in buffer (20 mM Tris pH 8.0, 150 mM NaCl, 5% glycerol) containing 1% Triton X-100.

After centrifugation, the supernatant was loaded on a Ni^2+^-NTA column (Qiagen, Hilden, Deutschland), equilibrated with buffer A (20 mM Tris, pH 8.0, 150 mM NaCl, 5% glycerol). The column was washed with five column-volumes (CV) of buffer A plus 10 mM imidazole and 0.1% Triton X-100, followed by 10 CV of buffer A containing 15 mM imidazole. Protein-His_6_ was eluted with 5CV of elution buffer (200 mM imidazole, pH 8.0, 5% glycerol, 1 mM dithiothreitol). Fractions containing the desired protein were dialyzed against Hepes buffer (50 mM, pH 7.0), concentrated to 5 mg/ml. Each fraction was analyzed by SDS-PAGE (Figure S2) and also was assayed for PNGase activity. Preparation and purification of PNGase F[36], Png1p and Png1p-ΔH1 from *Schizosaccharomyces pombe* [28] was done previously.

### Deglycosylation Assay

Stocks of 1 mg/ml Ribonuclease B (RNase B) was prepared in Hepes buffer (50 mM, pH 7.0). For glycoprotein denaturation, an aliquot from the stock was heated at 100°C for 10 min and rapidly frozen in a dry ice-ethanol bath. The structure was measured using J-810 Jasco spectropolarimeter (Jasco Co., Tokyo, Japan) as described previously [37]. 0.5 mg/ml RNase B was incubated with purified enzyme in 30 µl Hepes buffer (50 mM, pH 7.0) containing 5 mM DTT at 30°C. All proteins used in the assay were purified at the same time, following the same protocol. The molar ratio of the enzyme to substrate was 1∶30 in each reaction. After reaction, samples were analyzed by 15% SDS–PAGE followed by Coomassie staining. Quantification of deglycosylation was performed by densitometry with the Image J program.

### Molecular Modelling and Protein Domain Motion Analysis

The modeller module of Insight II 2005 was used to construct the molecular model of Png1p-ΔH1 from crystallographic structure of Png1p [1X3W][12]. The missing hydrogen atoms were added using the psfgen utility of NAMD [38]. The solvate script of VMD was used to place a cube of water around Png1p and Png1p-ΔH1 stretching 13 Å beyond the protein on all sides ions (50 mM NaCl) were added to ensure overall electrostatic neutrality. To mimic the stabilization effect of zinc binding, a Zn^2+^ was placed at the genomic center of four S atoms of the four Cys residues. The position of zinc atom and the four S atoms were all fixed during simulation.

NAMD with CHARMM 27 force field was used for all minimizations and molecular dynamic simulations [39]. A cut off of non-bonded and electrostatic interactions at 12 Å and switching between 10 and 12 Å was used. Electrostatic interactions were computed using the particle-mesh Ewald algorithm. The SHAKE algorithm was used to hold rigid the bond between each hydrogen atom and its mother atom. Each system was first minimized for 1000 cycles and was continued for another 1.75 ns equilibration with a time step of 2 fs at 298K. Trajectory data were collected at 0.5 ps intervals and subsequent analysis was performed using VMD.

### Surface Plasmon Resonance Analysis

Surface Plasmon Resonance (SPR) analysis was performed at 25°C using the UMPHOTM A400 SPR (Cytotrend, HK, China) in order to determine the substrate binding capability of Png1p and Png1p-ΔH1. For surface preparation, RNase B (40 µg/ml) in 10 mM sodium acetate buffer, pH 5.31, was immobilized on a CT405 sensor chip using standard amine coupling chemistry following the protocol recommended by the manufacturer. For kinetic analysis, duplicate injections of analytes (Png1p and Png1p-ΔH1) in various concentrations (80–1000 nM) were applied to the immobilized chip at a flow rate of 5 µl/min under the buffer condition of 10 mM HEPES, pH 7.4. The data obtained were analyzed with the SPRViewer software (version 1.0) provided by the manufacturer. A 1∶1 Langmuir model was globally fitted to the sensorgram data to obtain k_on_ and k_off_ values for the interaction. The equilibrium dissociation constant K_D_ was subsequently calculated as the ratio k_off_/k_on_.

### In-Gel Deglycosylation Analysis

Model glycoprotein RNase B separated by SDS-PAGE was stained with Coomassie blue. The targeted bands were excised and washed twice with NaHCO_3_ buffer (20 mM, pH 7.0) for 30 min. The washout was discarded and replaced by 300 µl of fresh NaHCO_3_ bufffer. Then 20 µl of 45mM dithiothreitol (DTT) was added and was incubated at 60°C for 30 min. The SDS and DTT were removed by incubation in 1∶1 acetonitrile/20 mM NaHCO_3_ buffer for 60 min. Subsequently, the excised bands were cut into smaller pieces of about 1 mm^3^ with a scalpel on a clean Petri dish. RNase B in gel were incubated with purified enzyme in 20 mM NaHCO_3_, pH 7.0[29]. All proteins used in the assay were purified at the same time, following the same protocol. After reaction, samples were analyzed by 15% SDS–PAGE followed by Coomassie staining. Quantification of deglycosylation was performed by densitometry with the Image J program.

## Supporting Information

Figure S1 Diagram of various Png1p deletion constructs.
(0.89 MB TIF)

Figure S2 Analysis of the Purified PNGase. Purified PNGase F, Png1p and Png1p-ΔH1 were subjected to SDS-PAGE followed by Coomassie staining. 1: Marker; 2: Png1p; 3: Png1p-ΔH1; 4: PNGase F.
(0.80 MB TIF)

Figure S3 Influence of temperature on Png1p activity. Png1p was incubated with denatured RNase B (0.5 mg/ml) in 30 µl of 50 mM Hepes buffer, pH 7.0 at different temperature for 1 h. All proteins used in the assay were purified at the same time, following the same protocol. The molar ratio of enzyme to substrate was 1∶30 in each reaction. Samples were taken at the indicated time points and subjected to SDS-PAGE followed by Coomassie staining. The zero time point was taken prior to addition of Png1p. The resulting Coomassie stained gels were quantified by densitometry with Image J program.
(0.83 MB TIF)

Figure S4 Comparison of the CαRMS deviation between Png1p (blue) and Png1p-ΔH1 (red).
(5.37 MB TIF)

Figure S5 Comparison of the CαRMS deviation over the last 200 ps for residues 33 to 62 in Png1p (blue) and Png1p-ΔH1 (red).
(6.68 MB TIF)

Table S1 Average RMSD of Helix H2, H3 and H4 before and after deletion
(0.03 MB DOC)

Table S2 Primers used in this study
(0.03 MB DOC)
