# Supplementary figures and images for: N-Terminal Deletion of Peptide:N-Glycanase Results in Enhanced Deglycosylation Activity
Source: PLoS One. 2009 Dec 16;4(12):e8335. doi: 10.1371/journal.pone.0008335 (PMC2791212; doi:10.1371/journal.pone.0008335)

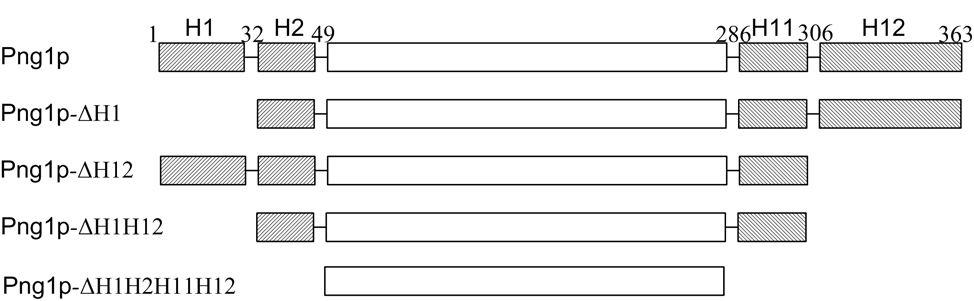

Supplement: Figure S1 — Diagram of various Png1p deletion constructs. (0.89 MB TIF) [file pone.0008335.s001.tif]

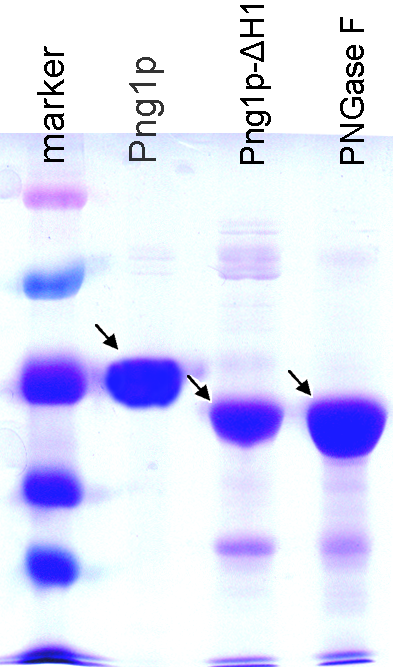

Supplement: Figure S2 — Analysis of the Purified PNGase. Purified PNGase F, Png1p and Png1p-ΔH1 were subjected to SDS-PAGE followed by Coomassie staining. 1: Marker; 2: Png1p; 3: Png1p-ΔH1; 4: PNGase F. (0.80 MB TIF) [file pone.0008335.s002.tif]

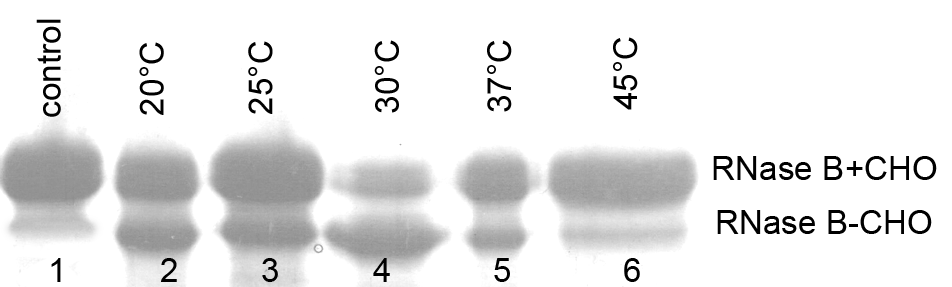

Supplement: Figure S3 — Influence of temperature on Png1p activity. Png1p was incubated with denatured RNase B (0.5 mg/ml) in 30 µl of 50 mM Hepes buffer, pH 7.0 at different temperature for 1 h. All proteins used in the assay were purified at the same time, following the same protocol. The molar ratio of enzyme to substrate was 1∶30 in each reaction. Samples were taken at the indicated time points and subjected to SDS-PAGE followed by Coomassie staining. The zero time point was taken prior to addition of Png1p. The resulting Coomassie stained gels were quantified by densitometry with Image J program. (0.83 MB TIF) [file pone.0008335.s003.tif]

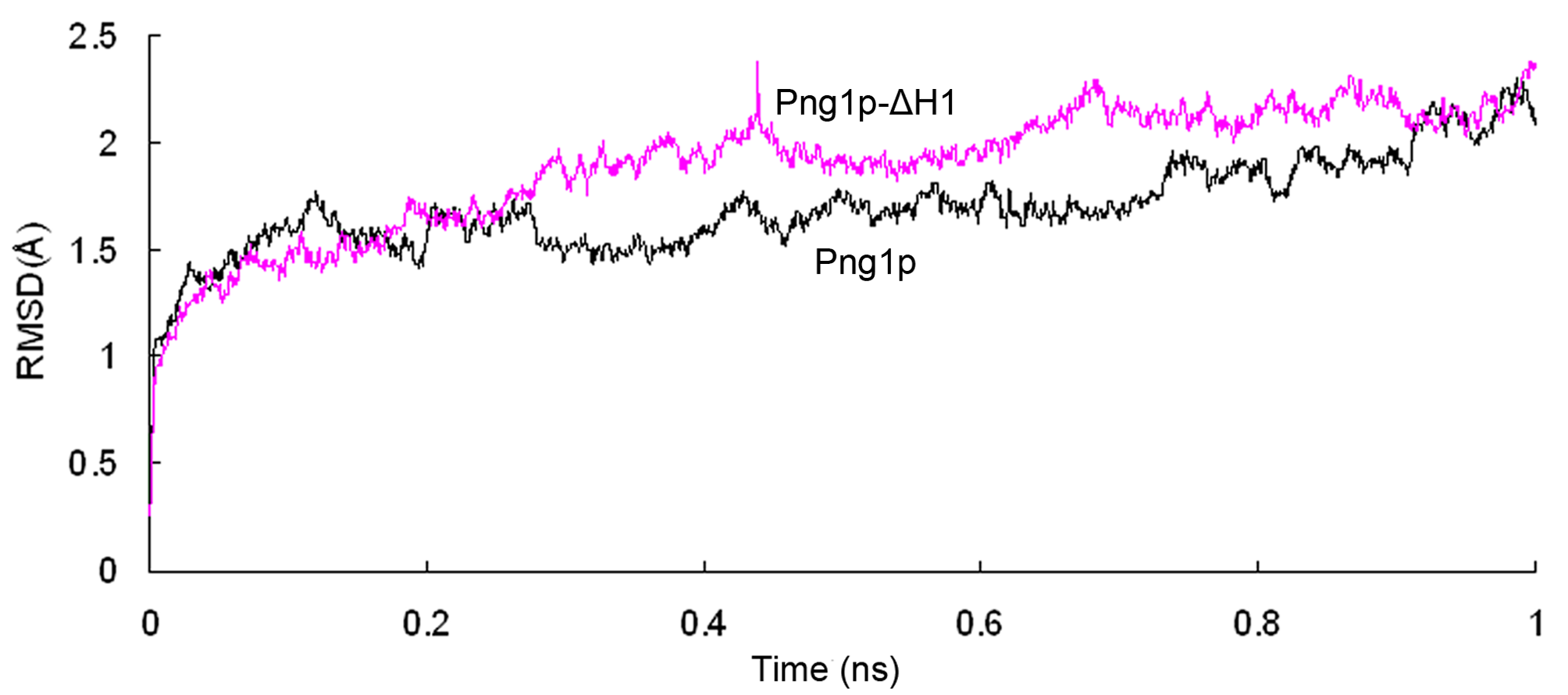

Supplement: Figure S4 — Comparison of the CαRMS deviation between Png1p (blue) and Png1p-ΔH1 (red). (5.37 MB TIF) [file pone.0008335.s004.tif]

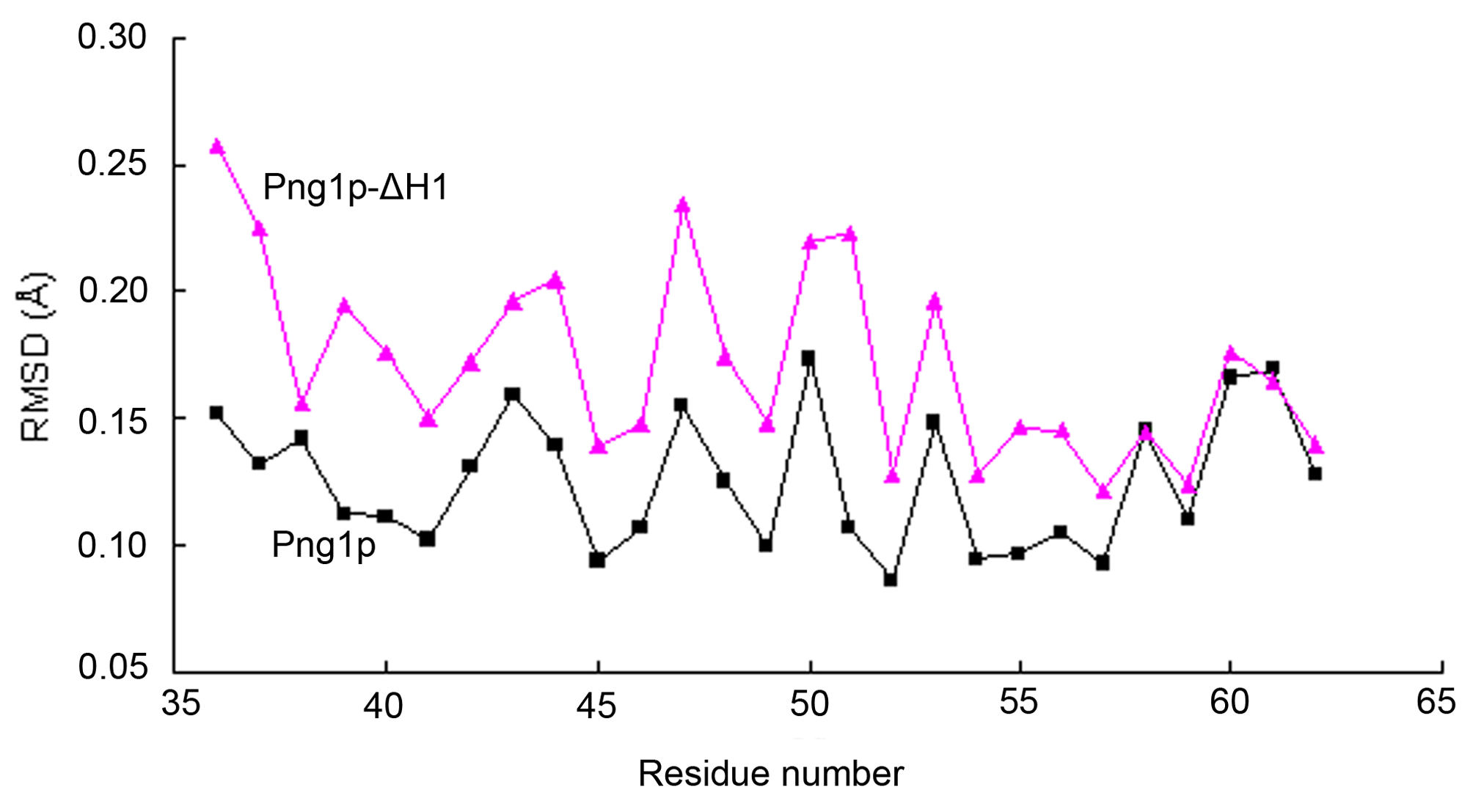

Supplement: Figure S5 — Comparison of the CαRMS deviation over the last 200 ps for residues 33 to 62 in Png1p (blue) and Png1p-ΔH1 (red). (6.68 MB TIF) [file pone.0008335.s005.tif]
