# Supplementary material for: N-Terminal Deletion of Peptide:N-Glycanase Results in Enhanced Deglycosylation Activity
Source: PLoS One. 2009 Dec 16;4(12):e8335. doi: 10.1371/journal.pone.0008335 (PMC2791212; doi:10.1371/journal.pone.0008335)
Supplement: Table S1 — Average RMSD of Helix H2, H3 and H4 before and after deletion (0.03 MB DOC) [file pone.0008335.s006.doc]

Table S1. Average RMSD of Helix H2, H3 and H4 before and after deletion

|  | Png1p | Png1p-∆H1 |
| --- | --- | --- |
| Helix 2  Helix 3  Helix 4 | 0.085  0.0672  0.0664 | 0.1021  0.0903  0.0893 |

Average RMSD is calculated over the final 200 ps of each simulation
