# Supplementary material for: N-Terminal Deletion of Peptide:N-Glycanase Results in Enhanced Deglycosylation Activity
Source: PLoS One. 2009 Dec 16;4(12):e8335. doi: 10.1371/journal.pone.0008335 (PMC2791212; doi:10.1371/journal.pone.0008335)
Supplement: Table S2 — Primers used in this study (0.03 MB DOC) [file pone.0008335.s007.doc]

Table S2 Primers used in this study

| primer | sequence |
| --- | --- |
| Png1 forward  Png1-∆H1 forward  Png1-∆H1, ∆H2 forward  Png1-∆H12 Reverse  Png1-∆H11,∆H12 Reverse  Png1 Reverse | 5’-TTGTGAATTCGATGGGAGAGGTATACGAAAA-3’ (*Eco*RI)  5 ’-TTGTGAATTCG AAAGCTGCACCTGTAGAGAA-3’ (*Eco*RI)  5’-TTGTGAATTCGCAATTCGCACCAAGGAGCTCT-3’ (*Eco*RI)  5 ’-TGTGTAAGCTTATTTAACGAATACGTCAACC-3’ (*Hin*dIII)  5’-TGTGTAAGCTTCTTGATTTGATCTCTGGGCA-3’ (*Hin*dIII)  5’-TGTTTAAGCTTTTTACCATCCTCCCCACGCT-3’ (*Hin*dIII) |
